# Functional characterization of the principal sigma factor RpoD of phytoplasmas *via* an *in vitro* transcription assay

**DOI:** 10.1038/srep11893

**Published:** 2015-07-07

**Authors:** Chihiro Miura, Ken Komatsu, Kensaku Maejima, Takamichi Nijo, Yugo Kitazawa, Tatsuya Tomomitsu, Akira Yusa, Misako Himeno, Kenro Oshima, Shigetou Namba

**Affiliations:** 1 Graduate School of Agricultural and Life Sciences, The University of Tokyo, 1-1-1 Yayoi, Bunkyo-ku, Tokyo, 113-8657, Japan; 2 Graduate School of Agriculture, Tokyo University of Agriculture and Technology, 3-5-8 Saiwaicho, Fuchu, Tokyo 183-8509, Japan

## Abstract

Phytoplasmas (class, *Mollicutes*) are insect-transmissible and plant-pathogenic bacteria that multiply intracellularly in both plants and insects through host switching. Our previous study revealed that phytoplasmal sigma factor *rpoD* of OY-M strain (*rpoD*_*OY*_) could be a key regulator of host switching, because the expression level of *rpoD*_*OY*_ was higher in insect hosts than in plant hosts. In this study, we developed an *in vitro* transcription assay system to identify RpoD_OY_-dependent genes and the consensus promoter elements. The assay revealed that RpoD_OY_ regulated some housekeeping, virulence, and host–phytoplasma interaction genes of OY-M strain. The upstream region of the transcription start sites of these genes contained conserved –35 and –10 promoter sequences, which were similar to the typical bacterial RpoD-dependent promoter elements, while the –35 promoter elements were variable. In addition, we searched putative RpoD-dependent genes based on these promoter elements on the whole genome sequence of phytoplasmas using *in silico* tools. The phytoplasmal RpoD seems to mediate the transcription of not only many housekeeping genes as the principal sigma factor, but also the virulence- and host-phytoplasma interaction-related genes exhibiting host-specific expression patterns. These results indicate that more complex mechanisms exist than previously thought regarding gene regulation enabling phytoplasmas to switch hosts.

In the regulation of bacterial gene expression, the initiation of transcription, mediated by a DNA-dependent RNA polymerase (RNAP) holoenzyme, plays an important role as the first step in the regulation process. The RNAP holoenzyme consists of a core enzyme (subunit composition α2ββ'ω) with catalytic activity of RNA polymerization, and an additional subunit known as a sigma factor involved in promoter recognition and DNA melting^1^. Most bacteria have multiple sigma factors that recognize different sets of promoters as key regulators of stress responses to environmental changes and basal gene expression. RpoD (also known as sigma 70) is the principal (primary) and well-studied sigma factor responsible for the transcription of housekeeping genes in most bacteria^1^. The intracellular concentration of RpoD in *Escherichia coli* is maintained at a constant level under various growth conditions^2^. The sigma 70 family proteins, including RpoD, contain four conserved regions designated as 1 to 4^3^. In general, two DNA binding domains that reside in regions 2 and 4 recognize conserved promoter hexamer sequences (promoter elements) around positions approximately 10 and 35 nucleotides upstream, respectively, of the transcription start sites (TSSs)^1^. Previous studies have revealed that in many bacteria, RpoD interacts with the two archetypal promoter elements (–35 5′-TTGACA-3′ and –10 5′-TATAAT-3′ separated by an about 17-bp spacer)^4^.

Phytoplasmas (class *Mollicutes*, genus ‘*Candidatus* Phytoplasma spp.’) infect hundreds of plant species and cause devastating yield losses in various crops worldwide^5^,^6^. Phytoplasmas are biologically unique in that they can parasitize both plants (Kingdom Plantae) and insects (Kingdom Animalia). Although studies have shown that some phytoplasmas are vertically transmitted at low rates to plant embryos or insect progeny, they depend completely on “host switching” between plants and insects for their survival and dispersal^7^,^8^. Phytoplasmas dramatically alter their gene expression in response to host switching. For example, *tengu* and *phyl1*, phytoplasma genes encoding a secreted protein, are more highly expressed in plant hosts than insect hosts, and respectively induce phytoplasma-specific symptoms in plants such as witches’ broom and phyllody^9^,^10^. Moreover, we previously showed that at least 33% of the genes in the genome of ‘*Ca.* P. asteris’ onion yellows strain (OY-M) are differentially expressed when grown in plant versus insect hosts^11^.

Although the alternation of gene expression in response to host switching is assumed to be important for the host adaptation of phytoplasmas, how they regulate their gene expression to adapt to the two distinct intracellular environments (i.e., plant and insect cells) is unclear. To date, only limited studies have addressed the gene regulatory mechanisms in phytoplasmas due to the difficulties associated with their genetic engineering. All five genome-sequenced strains of phytoplasma have two types of sigma factors, RpoD and FliA^12^,^13^,^14^,^15^,^16^. Phytoplasmal RpoD, which has high amino acid sequence similarity with RpoD of *E. coli*, is highly conserved in each of the phytoplasma genomes as a single-copy gene (see Supplementary Fig. S1 online). Phytoplasmal RpoD contains all four conserved regions typical for sigma 70-type sigma factors. An amino acid sequence alignment of the RpoD homologous proteins shows that their C-terminal half, which contains regions 2 to 4 largely conserved in sigma factors, is almost identical to each other, whereas the N-terminal half containing region 1 was less conserved (see Supplementary Fig. S2 online). Given that regions 2 and 4 of RpoD are involved in the recognition of –10 and –35 promoter elements, respectively^1^, RpoDs of each phytoplasma likely have similar promoter recognition specificity. However, phytoplasmal *fliA* has detectable sequence similarity with an alternative sigma factor sigma 28 of *E. coli* or the extracytoplasmic function (ECF) sigma factor subfamily and is present in each of the phytoplasma genomes as a multi-copy gene, with the exception of ‘*Ca*. P. mali’; these FliAs encode proteins containing only region 2, or regions 2 and 4 (see Supplementary Fig. S3 online)^12^,^13^,^14^,^15^,^16^.

The genome of OY-M contains 13 copies of putative sigma factor genes, one of which is categorized as *rpoD* (PAM_628; *rpoD*_*OY*_) and the others are categorized as *fliA* ( *fliA*_*OY*_). Despite the role of RpoD in *E. coli* and many other bacteria in regulating housekeeping genes^17^,^18^, the expression level of *rpoD* of OY-M (*rpoD*_*OY*_) is 4.0 times higher in insect hosts than in plant hosts^11^,^19^, even though actual accumulation level of RpoD_OY_ protein is unknown. These data raise doubts as to whether RpoD_OY_ functions as the principal sigma factor. A few studies have approached this issue using *ex vivo* or *in silico* methods. A recent study using an *E. coli*-based *ex vivo* reporter assay (EcERA) system that evaluates the interaction between phytoplasmal promoters and sigma factors based on the luciferase reporter activity in *E. coli* cells showed that RpoD_OY_ activates the promoter regions (approximately 400 bp) of several housekeeping genes highly expressed in insect hosts^19^. These results suggest that RpoD_OY_ has the potential to function as the principal sigma factor and plays a major role in infected insect hosts. However, due to possible indirect effects of cognate transcription factors in living *E. coli* cells, avoiding false positives and negatives in this EcERA system was difficult. Thus, this system does not appear to be suitable for further research to identify promoter elements recognized by RpoD_OY_. In another study, bioinformatic prediction using *E. coli* RpoD-dependent promoter elements was performed to identify phytoplasma RpoD-dependent promoters. However, obtaining precise estimates using this approach is difficult, since phytoplasma genomes, as well as *E. coli* RpoD-dependent promoter elements, are strongly AT-biased^4^,^20^. These results indicate that novel approaches are needed to provide more accurate measurements of the promoter activity regulated by RpoD_OY_ to determine its function.

In this study, to elucidate the role of RpoD_OY_ in phytoplasma gene expression in detail, we developed an *in vitro* transcription assay of phytoplasma genes and analyzed their promoter activity using this assay. The consensus RpoD_OY_-dependent promoter elements were identified for the first time. The genome-wide promoter prediction using this newly identified essential promoter elements revealed at least 88 genes that were regulated by RpoD_OY_, which was involved in the transcription of not only many housekeeping genes, but also virulence- and host-phytoplasma interaction-related genes. We discuss the mechanism of transcription regulation associated with host switching between plants and insects based on the function of RpoD_OY_.

## Results

### RpoD_OY_ recognizes two *rrnB* promoters

**Figure 1:**
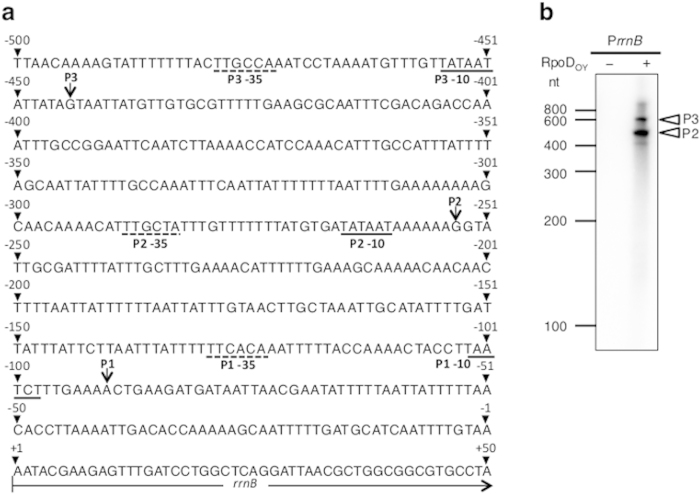
Identification of *rrnB* TSSs and analysis of *rrnB* promoter activity based on the *in vitro* transcription assay. (**a**) Schematic representation of the upstream promoter region and TSSs of the *rrnB* gene. The *rrnB* TSSs identified by 5′ RACE analysis, designated as P1, P2, and P3 (91, 254, 444 nt upstream of *rrnB*, respectively), are represented by arrows. Putative –35 and –10 promoter elements of each of the three TSSs are underlined with dotted and continuous lines, respectively. (**b**) *In vitro* transcription assays using the RNAP holoenzyme with RpoD_OY_. RNAP_Ec_ and a DNA template were incubated with NTP, including [γ-32P]CTP in the absence (–) or presence (+) of RpoD_OY_. A 784-bp DNA fragment named P*rrnB* covering the region from –500 to +284 of *rrnB* was used as a template. White arrowheads indicate the positions of the transcripts that are possibly transcribed from P2 and P3.

RpoD recognizes specific promoter elements located at positions 35 and 10 bp upstream of TSSs^1^, and hence, an experimental determination of TSSs provides important clues in estimating their upstream promoter elements. For the purpose of identifying promoter elements recognized by RpoD_OY_, we investigated the TSSs of the 16S ribosomal RNA (*rrn*) B gene of OY-M, as *rrn* is often transcribed from RpoD-dependent promoter elements in many bacteria^21^,^22^,^23^,^24^. A 5′ rapid amplification of cDNA ends (5′ RACE) analysis of *rrnB* was performed using total RNA from phytoplasma-infected plants, resulting in the detection of three TSSs located 91, 254, and 444 bp upstream of *rrnB* (Fig. 1a). The same results were obtained from total RNA extracted from phytoplasma-infected insects. We designated these TSSs as P1, P2, and P3, respectively, and estimated their putative promoter elements as follows: P1 promoter elements, –35 5′-TTCACA-3′ and –10 5′-TAATCT-3′; P2 promoter elements, –35 5′-TTGCTA-3′ and –10 5′-TATAAT-3′; and P3 promoter elements, –35 5′-TTGCCA-3′ and –10 5′-TATAAT-3′. Among these promoter elements, putative P2 and P3 promoter elements were highly similar to each other.

To identify which of these putative *rrnB* promoter elements are recognized by RpoD_OY_, we performed *in vitro* transcription assays, which have been used in studies of bacterial transcription systems. Some examples of the assays include a study identifying core promoter elements in a given input DNA sequence and then trying to correlate these elements to DNA-binding proteins such as sigma factors^25^. For *in vitro* transcription assays using RpoD_OY_, we reconstituted the RNAP holoenzyme with a commercially available *E. coli* RNAP core enzyme (RNAP_Ec_) and purified recombinant RpoD_OY_ (RNAP_Ec_–RpoD_OY_; see Supplementary Fig. S4 online). A 784-bp DNA fragment from –500 to +284 of *rrnB*, named P*rrnB* and covering the three identified TSSs (P1, P2, and P3) and their putative promoter regions, was used as a template for transcription reactions. As shown in Fig. 1b, two major transcripts of approximately 500 and 700 nt were observed in an RpoD_OY_-dependent manner. The sizes of these transcripts corresponded to the expected sizes of transcripts initiated from P2 (254 bp upstream of *rrnB*) and P3 (444 bp upstream of *rrnB*), respectively. No transcripts corresponding to that transcribed from P1 (91 bp upstream of *rrnB*), which was expected to be 375 nt, were detected. These results indicate that RpoD_OY_ is compatible with the heterologous *E. coli* RNAP to initiate transcription as the RNAP_Ec_–RpoD_OY_ holoenzyme. Our results also demonstrated that the RNAP holoenzyme with RpoD_OY_ recognizes the *rrnB* promoter elements upstream of P2 and P3, but not P1.

### Identification of the *rrnB* core promoter sequence recognized by RpoD_OY_

**Figure 2:**
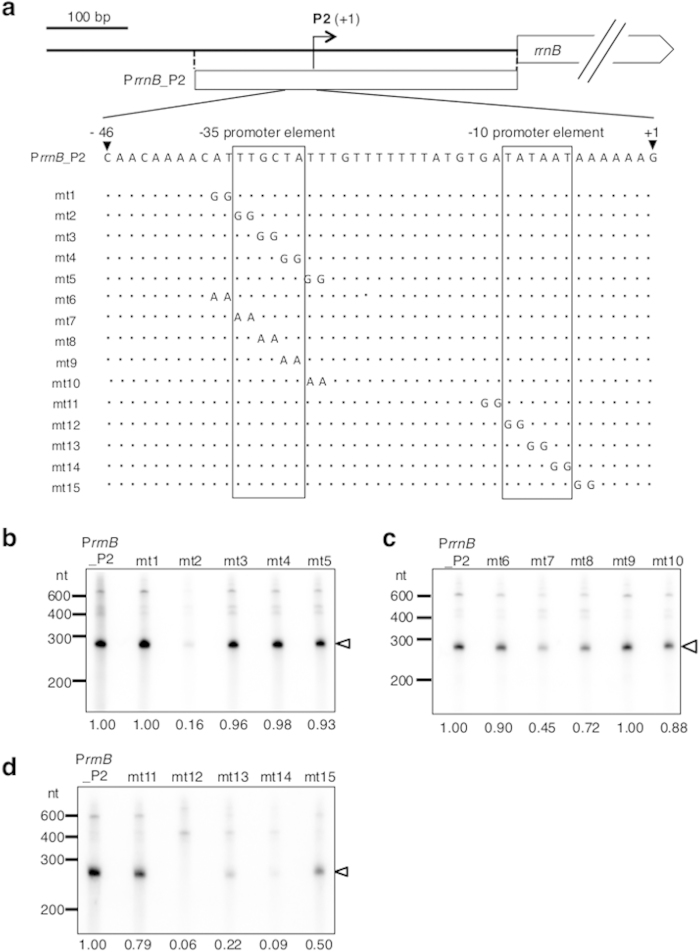
Effects of nucleotide substitutions in the putative *rrnB* promoter elements. (**a**) Schematic representation of mutant templates with substitutions in the putative P2 promoter elements. The open box represents a 400-bp DNA fragment covering the region from 400 to 1 nt upstream of *rrnB*, which was used as a control template (P*rrnB*_P2). A series of 15 mutant templates were prepared, with substitutions to GG or AA introduced around the putative P2 –35 promoter element (mt1, mt2, mt3, mt4, and mt5; mt6, mt7, mt8, mt9, and mt10) or around the putative P2 –10 promoter element (mt11, mt12, mt13, mt14, and mt15). ‘+1’ represents the P2 TSS. Positions of putative P2 –35 and –10 promoter elements are boxed. Dots indicate the same sequence as P*rrnB*_P2. *In vitro* transcription with templates containing substitutions to GG (**b**) or AA (**c**) in the putative P2 –35 promoter element, and substitutions to GG in the putative P2 –10 promoter element (**d**). RNAP_Ec_–RpoD_OY_ holoenzyme and DNA templates were incubated with NTP including [γ-32P]CTP. White arrowheads indicate the positions of transcripts from each template. Numbers at the bottom of each *in vitro* transcription lane represent the relative quantification of autoradiography signals measured by using ImageJ software (version 1.47, National Institutes of Health).

To define the core promoter elements of *rrnB*, we focused on the putative P2 promoter and performed *in vitro* transcription assays. We introduced a series of double-base substitutions to GG into the putative P2 –35 and –10 promoter elements of a DNA fragment containing –400 to –1 of *rrnB*, named P*rrnB*_P2 (Fig. 2a). In the putative –35 promoter element of P2, the double-base substitution of TT to GG at positions –35 and –34 (mt2) resulted in a drastic reduction of the transcript level to 16% compared with the intact P*rrnB*_P2 (Fig. 2b), suggesting decreases in activity of the putative P2 promoter due to these mutations. In contrast, nucleotide substitutions at positions –37 and –36 (mt1), or –33 to –28 (mt3, mt4, and mt5), did not alter promoter activity. Similar results were obtained with the substitutions of TT to AA at the same positions (Fig. 2c). These results suggest that TT at positions –35 and –34 is crucial for promoter activity mediating transcription from *rrnB* P2. In the P2 –10 promoter element, the substitutions at positions from –12 to –7 (mt12, mt13, and mt14) also resulted in a reduction in promoter activity to 6–22% compared with the intact P*rrnB*_P2 (Fig. 2d). Substitutions at positions –14 and –13 (mt11), and –6 and –5 (mt15), of P2 slightly decreased promoter activity (Fig. 2d). These results suggest that the conserved hexamer, 5′-TATAAT-3′, is crucial for promoter activity. These findings indicate that at a minimum, the upstream sequence 5′-TT-21bp-TATAAT-3′ of *rrnB* P2 is essential for recognition by RpoD_OY_. This is supported by the fact that the same sequence exists in the putative P3 promoter elements (–35 5′-TTGCCA-3′ and –10 5′-TATAAT-3′), but not in the putative P1 promoter elements (–35 5′-TTCACA-3′ and –10 5′-TAATCT-3′).

### RNAP holoenzyme containing RpoD_OY_ mediates the transcription of various categories of genes

**Figure 3:**
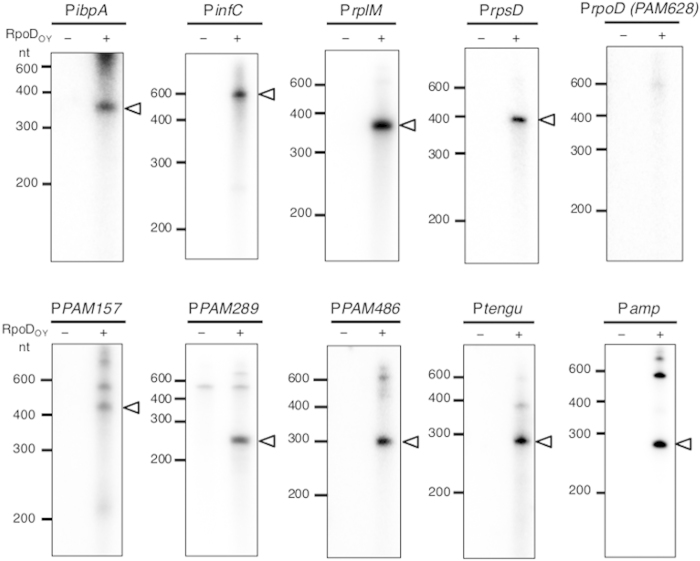
*In vitro* transcription with templates containing the promoter region of various gene categories. RNAP_Ec_-RpoD_OY_ and DNA templates were incubated with NTP, including [γ-32P]CTP in the absence (–) or presence (+) of RpoD_OY_. The length of each template is as follows: P*ibpA*, 713 bp (–512 to +201 of *ibpA*); P*infC*, 702 bp (–500 to +202 of *infC*); P*rplM*, 700 bp (–500 to +200 of *rplM*); P*rpsD*, 650 bp (–450 to +200 of *rpsD*); P*rpoD*, 624 bp (–400 to +224 of *rpoD*); P*157*, 500 bp (–300 to +200 of *PAM157*); P*289*, 500 bp (–500 to –1 of *PAM289*); P*486*, 566 bp (–300 to +266 of *PAM486*); P*tengu*, 420 bp (–300 to +120 of *tengu*); P*amp*, 400 bp (–200 to +200 of *amp*). White arrowheads indicate the positions of transcripts from each template.

To investigate the OY-M genes regulated by RpoD_OY_, we performed *in vitro* transcription assays using other templates. Many other bacteria housekeeping genes have sigma 70-type promoters^26^,^27^,^28^,^29^, so we used the upstream regions of four housekeeping genes of OY-M [the protein chain initiation factor IF-3 (*infC*), 50S ribosomal subunit protein L13 (*rplM*), 30S ribosomal subunit protein S4 (*rpsD*), and RNA polymerase sigma70 factor (*rpoD*) genes] for *in vitro* transcription as templates (P*infC*, P*rplM*, P*rpsD*, and P*rpoD*, respectively). In addition, the upstream region of the molecular chaperone gene (*ibpA*) was used as a template (P*ibpA*) because a previous study suggested that it is regulated by RpoD in an AT-rich bacterium ‘*Ca.* Blochmannia floridanus’^30^, while in some other bacteria, *ibpA* is reported to be regulated by an alternative heat shock sigma factor RpoH^31^,^32^. Our *in vitro* transcription assays revealed that RNAP_Ec_–RpoD_OY_ produced specific transcripts from the four templates (P*infC*, P*rplM*, P*rpsD*, and P*ibpA*), but did not produce a transcript from P*rpoD* (Fig. 3). When only RNAP_Ec_, which lacks RpoD_OY_, was added to the reaction, no specific transcripts were observed from any of these templates (Fig. 3). These results indicate that RpoD_OY_ mediates the transcription of many phytoplasma housekeeping genes, although with some exceptions. Considering the previous findings that RNAP containing the principal sigma factor transcribes the majority of the housekeeping genes^3^, RpoD_OY_ is likely to play a role as the principal sigma factor.

To examine whether RNAP_Ec_-RpoD_OY_ recognizes OY-M gene promoters other than housekeeping genes, we performed *in vitro* transcription assays using five additional templates (P*PAM157*, P*PAM289*, P*PAM486*, P*tengu*, and P*amp*) containing upstream regions of genes associated with virulence or host–phytoplasma interactions: *PAM157* (putative secreted protein), *PAM289* (adhesin-like protein)^33^, *PAM486* (putative secreted protein), *tengu* (secreted and virulence-related protein)^9^, and *amp* (insect transmissibility-related protein)^34^. In previous studies, we reported that *PAM157* and *PAM289* were highly expressed in insect hosts and *PAM486* and *tengu* were highly expressed in plant hosts^9^,^11^, while *amp* was expressed at comparable levels in both plant and insect hosts^11^. The *in vitro* transcription assays revealed that RNAP_Ec_–RpoD_OY_ produced specific transcripts from all templates, P*PAM157*, P*PAM289*, P*PAM486*, P*tengu*, and P*amp* (Fig. 3). These results suggest that an RNAP holoenzyme containing RpoD_OY_ recognizes not only housekeeping genes, but also the genes related to virulence and host–phytoplasma interactions.

### Identification of consensus RpoD_OY_-dependent promoter elements

**Figure 4:**
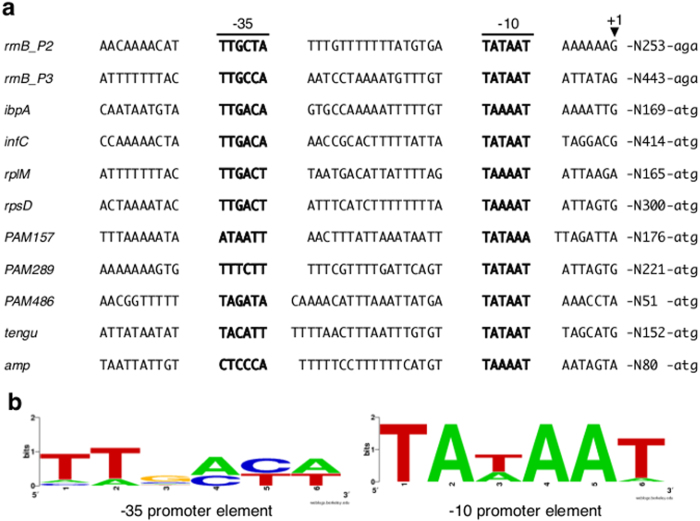
Sequence conservation in the RpoD_OY_-dependent promoter region. (**a**) Putative promoter sequences deduced from the alignment of upstream sequences of TSSs identified by 5′ RACE analysis. Bold letters indicate the putative –35 and –10 promoter elements. ‘+1’ represents the position of the TSS. Numbers of the right side show the distance to the 5´-end of mature rRNA or ATG. (**b**) Consensus sequences of RpoD_OY_-dependent promoters. The consensus sequences of –35 (left) and –10 (right) promoter elements were determined using the BioProspector program^63^ and illustrated with the WebLogo tool^64^.

To more closely define the RpoD_OY_-dependent promoter elements, we performed a 5′ RACE analysis of nine genes shown in this study to be transcribed by RNAP_Ec_–RpoD_OY_ (Fig. 3; *ibpA*, *infC*, *rplM*, *rpsD*, *PAM157*, *PAM289*, *PAM486*, *tengu*, and *amp*) using total RNA from phytoplasma-infected plants or insects. As a result, we mapped the 5′-ends of *ibpA*, *infC*, *rplM*, *rpsD*, *PAM157*, *PAM289*, *PAM486*, *tengu*, and *amp* transcripts at 170, 415, 166, 301, 177, 222, 52, 153, and 81 nt upstream, respectively, of their start codons (Fig. 4a). In agreement with a previous study on the detection and identification of mycoplasma promoter sequences^35^, the 5′-end of these transcripts was either adenine or guanine. The predicted size from these identified TSSs to the 3′-ends of the above-mentioned *in vitro* transcription templates roughly corresponded to the length of transcripts produced by the *in vitro* transcription assays (Fig. 3). Next, to further characterize the RpoD_OY_-dependent promoter elements, we searched for consensus promoter elements using a motif finding tool, BioProspector, and found conserved –35 and –10 hexamers located at appropriate positions upstream of the TSSs (Fig. 4a). The consensus –10 promoter element (5′-TAtAAT-3′) was found in all sequences examined (Fig. 4b). The consensus –35 promoter element (5′-TTgaca-3′) was also found, even though this element was less conserved compared with the –10 promoter element (Fig. 4b). The spacing between the –35 and –10 promoter elements could vary from 17 to 19 nt. We also found two relatively conserved regions, an ‘extended –10 motif’ (5′-TnTG-3′) positioned around –17 to –14 and an A-rich region positioned around –42 to –39 (see Supplementary Fig. S5 online), which are common features of the promoter regions in other bacteria^36^,^37^.

### Genome-wide prediction of RpoD_OY_-dependent genes

Based on the consensus RpoD_OY_-dependent promoter elements identified in this study with several sequence variants (Fig. 4b; [TC][AT][GC][AC][TC][AT]N_17–19_TA[AT]AA[AT]), we searched putative RpoD_OY_-dependent genes that possess these promoter elements on the whole genome sequence of the OY-M phytoplasma^12^ using a DNA motif-finding program, RSA-tools facilities. Among a total of 540 sequence hits, we found 103 putative RpoD_OY_-dependent promoter elements located within a 500-bp upstream region of either ATG initiation codons or the 5′-ends of mature tRNA and rRNA of OY-M genes, which could mediate the transcription of at least 88 genes (about 12% of all OY-M genes; see Supplementary Table S3 online). Among these 103 putative promoters, twenty-two promoters (21.4%) contained a 5′-TG-3′ motif at the extended –10 region, including seventeen promoters (16.5%) with the 5′-TnTG-3′ motif. These results agree with a previous study of *E. coli* promoters^38^. Subsequently, these 88 putative RpoD_OY_-dependent genes were classified into Clusters of Orthologous Groups^39^ based on their predicted functions, which showed that 25 genes (29%) belong to the category of information storage (replication, transcription, and translation), and 11 genes (12%) and 8 genes (9%) belong to the category of metabolism (e.g., ABC-transporters) and cellular processes (e.g., co-chaperonin and zinc proteases), respectively (see Supplementary Fig. S6 online). Previous studies have suggested that phytoplasma genes that are highly expressed in insect hosts are likely to be regulated by RpoD_OY_, which is also highly expressed in insect rather than plant hosts^11^,^19^. However, we did not find a correlation between the RpoD_OY_ dependence of phytoplasma genes predicted in this study and their host-specific expression pattern during host switching as described in our previous microarray analysis^11^. According to the microarray results, among the 88 putative RpoD_OY_-dependent genes, as many as 32 genes showed no significant differences in expression pattern between plant and insect hosts, while 21 and 10 genes were upregulated more than twofold in plant and insect hosts, respectively.

To examine whether RpoD homologous proteins in other species of phytoplasma also regulate similar sets of genes such as OY-M, an *in silico* promoter analysis was carried out using the whole genomes of the three phytoplasmas [‘*Ca*. P. asteris’ strain AYWB (AYWB), ‘*Ca*. P. australianse’ (PAa), and ‘*Ca*. P. mali’ strain AT (ATP)]. Given that RpoD homologous proteins of these phytoplasmas contain highly conserved motifs, regions 2 and 4, which are responsible for the recognition of the –10 and –35 promoter elements, respectively (see Supplementary Fig. S2 online), their promoter recognition specificity would likely be similar to that of RpoD_OY_. Therefore, we used the same query sequences ([TC][AT][GC][AC][TC][AT]N_17–19_TA[AT]AA[AT]) for promoter prediction. In total, 68, 71, and 100 putative RpoD-dependent genes were found in AYWB, PAa, and ATP, respectively. Of the 88 putative RpoD_OY_-dependent genes in OY-M, 34 genes (39%) were also predicted to be RpoD-dependent in at least one of the other phytoplasma strains. Various housekeeping genes such as ribosomal RNA genes, ribosomal protein subunit genes, and tRNAs, were found in common among the putative RpoD-dependent genes of all three phytoplasmas (see Supplementary Table S3 online), implying that phytoplasmal RpoD plays a role, at least in part, as the principal sigma factor.

## Discussion

All known eubacteria possess the principal sigma factor responsible for transcription of the majority of housekeeping genes. Hence, identification of the promoter elements recognized by the principal sigma factor is an important step toward understanding gene regulation mechanisms in bacteria. The principal sigma factors in culturable bacteria, such as *E. coli* RpoD and *Bacillus subtilis* SigA, have been well described, and their target promoter elements have been intensively studied genetically^22^,^38^,^40^. However, little is currently known about the sigma factors and their promoter sequences of obligate parasitic bacteria, including phytoplasmas, due to the difficulty of their *in vitro* culture and genetic engineering. In this study, we identified the RpoD-dependent promoters in OY-M phytoplasma by the development of an *in vitro* transcription assay system using an RNAP holoenzyme heterologously reconstituted with RNAP_Ec_ and RpoD_OY_. This system would be also a powerful tool for studying gene regulatory mechanisms of other uncultured bacteria.

The *in vitro* transcription system has the advantage that transcripts synthesized from each promoter can be discriminated by their lengths. Based on the lengths of two major *in vitro* transcripts from P*rrnB*, which was the DNA template containing the putative *rrnB* promoter region (Fig. 1b), they were considered to be transcribed from promoter elements in the upstream region of P2 and P3 that were two out of the three *rrnB* TSSs determined by 5′ RACE analysis (Fig. 1a). These putative promoter elements upstream of P2 (–35 5′-TTGCTA-3′ and –10 5′-TATAAT-3′) and P3 (–35 5′-TTGCCA-3′ and –10 5′-TATAAT-3′) were similar to the typical bacterial RpoD-dependent core promoter elements (–35 5′-TTGACA-3′ and –10 5′-TATAAT-3′; Fig. 1a). Our results agree with earlier observations that in many bacteria, the upstream region of *rrn* has promoter elements that can be recognized by RpoD^23^,^24^. In particular, similar to *rrnB* of phytoplasmas, the *rrn* of *E. coli* and *B. subtilis* has two sets of promoter elements, which can also be recognized by sigma 70-type sigma factors^21^,^22^. However, the *rrnB* P1 promoter might be under the control of a sigma factor other than RpoD_OY_, since no transcript corresponding to those from P1 was detected in the *in vitro* transcription system employing the RNAP_Ec_–RpoD_OY_ holoenzyme despite the fact that, *in vivo*, we identified the transcripts from P1 *via* the 5′ RACE analysis. We also found nine genes (*ibpA*, *infC*, *rplM*, *rpsD*, *PAM157*, *PAM289*, *PAM486*, *tengu*, and *amp*) that have only one TSS with –10 and –35 promoter elements resembling the RpoD_OY_-dependent promoters of *rrnB* (P2 and P3) (Fig. 4a). Moreover, the predicted size from these identified TSSs to the 3′-ends of the *in vitro* transcription templates roughly corresponded to the length of transcripts in the RpoD_OY_-mediated *in vitro* transcription assays (Fig. 3). These results indicate that these genes are also most likely RpoD_OY_-dependent. If there were other sigma factors that transcribe these genes, two or more 5′ end of *in vivo* transcripts would be identified.

Among them, three genes (*rrnB*, *PAM289*, and *tengu*) were common between the *in vitro* transcription assay in this study and another *in vivo* promoter assay^19^. The upstream regions of *rrnB* and *PAM289* were recognized by RNAP_Ec_-RpoD_OY_ in our *in vitro* transcription assay (Fig. 3), which is consistent with the results of the EcERA system^19^. The *tengu* promoter recognized by RpoD_OY_ in our *in vitro* transcription (Fig. 3), however, exhibited no significant increases in transcription activity by RpoD_OY_ in the EcERA system^19^. Our 5′ RACE analysis using total RNA derived from phytoplasma-infected plants showed that the upstream region of major TSSs of *tengu* actually contains typical RpoD_OY_-dependent promoter elements (–35 5′-TACATT-3′ and –10 5′-TATAAT-3′ ; Fig. 4a), suggesting that *tengu* is also regulated *in vivo* by RpoD_OY_. Thus, the results of our *in vitro* transcription assay were not necessarily consistent with those obtained from the EcERA system. A possible explanation for this discrepancy between our results and those from the previous study is the side effects of several positive and negative transcriptional regulators of *E. coli* in the EcERA system. These regulators may affect the results of EcERA due to the interaction between these regulators of *E. coli* and target promoter sequences of phytoplasmas. Moreover, overexpression of RpoD_OY_ can perturb the expression pattern of these regulators of *E. coli* because we revealed that RpoD_OY_ recognizes promoter elements similar to those recognized by *E. coli* RpoD. Therefore, although promoters that require activation by other transcriptional regulators are likely to escape detection by our *in vitro* transcription system, it seems to be a more accurate tool compared to the EcERA system in measuring the specific activity of promoters recognized by RpoD_OY_.

Our *in vitro* transcription assay mediated by RpoD_OY_ revealed that nucleotide substitutions in the –10 promoter element of *rrnB* P2 drastically influenced promoter activity (Fig. 2d). In contrast, substitutions in the –35 promoter element, with the exception of the TT motif on the 5′-side, had little effect on promoter activity (Fig. 2b,c). Moreover, the –35 promoter elements of RpoD_OY_-dependent phytoplasma genes were highly variable (Fig. 4b). In agreement with these findings, among the RpoD-dependent promoters of some other bacteria such as *E. coli*, *Campylobacter jejuni*, and *Mycoplasma hyopneumoniae*, the –10 promoter elements are very similar to each other, but the –35 promoter elements are relatively variable^4^,^35^,^41^. Nucleotide substitutions in the –35 promoter element of *Chlamydia trachomatis*, an obligate intracellular pathogen, had smaller effects compared to substitutions in the –10 promoter element on the recognition of the RNAP holoenzyme^24^. In addition, in *M. hyopneumoniae*, which has a small AT-rich genome similar to phytoplasmas, no obvious –35 promoter elements were identified upstream of the TSSs of each gene, while the typical –10 promoter elements (5′-TATAAT-3′) were found^35^. Thus, sequence features of the RpoD_OY_-dependent promoter elements identified in this study were consistent with previous observations of other bacteria.

In addition to the –35 and –10 promoter elements, the RpoD_OY_-dependent promoter possessed other sequence features common in bacteria, such as a 5′-TnTG-3′ positioned around –17 to –14 (“the extended –10 region”) and an A-rich region positioned around –42 to –39 (see Supplementary Fig. S5 online)^36^,^37^. In *E. coli*, the extended –10 region and the A-rich region were suggested to interact with region 3.0 (previously named 2.5) of RpoD and the α-subunit of RNAP, respectively^42^,^43^. In the region 3.0, histidine and glutamic acid residues, which are conserved among bacterial sigma factors including phytoplasmal RpoDs (see Supplementary Fig. S2 online), are involved in contacting the extended -10 region^42^. Nucleotide substitution of the extended –10 region resulted in a drastic reduction of the promoter activity in *B. subtilis* and *E. coli*^38^,^40^. Moreover, in *C. trachomatis*, nucleotide substitutions at positions 4 and 5 bp upstream of TSSs, where no conserved motif has been identified, had negative effects on promoter activity^24^. Our *in vitro* transcription assay using RpoD_OY_ revealed that nucleotide substitutions at positions –14 and –13 (mt11), and –6 and –5 (mt15), slightly decreased promoter activity (Fig. 2d). Examining the contribution of these extended regions of the phytoplasma promoter to transcriptional activity should be interesting.

In many bacteria, various sigma factors compete for a limited amount of RNAP core enzyme^2^, and the ratio of individual sigma factors could affect gene expression patterns. In general, the intracellular concentration of the principal sigma factor RpoD is held constant under ordinary conditions for transcription of the majority of the housekeeping genes, and other alternative sigma factors are transiently expressed under specific conditions for bacterial adaptation to environmental changes^2^,^26^. However, *rpoD*_*OY*_ expression is approximately four times more abundant in insect hosts compared to plant hosts in the phytoplasma life cycle^11^,^19^, while the expression level of *fliA*_*OY*_, another type of sigma factor gene in OY-M, does not differ significantly between the two host types^19^.

We had initially hypothesized that RpoD_OY_ would regulate the expression of genes that are highly expressed in insect hosts as the alternative sigma factor rather than the principal sigma factor^11^. Contrary to this hypothesis, our *in silico* search for RpoD_OY_-dependent genes suggested that RpoD_OY_ mediated the transcription of many housekeeping genes as the principal sigma factor (see Supplementary Table S3 and Fig. S6 online). In addition, our *in vitro* transcription assays revealed that RpoD_OY_ recognized the promoters of *tengu* and *PAM486* (Fig. 3), which are highly expressed in plant hosts. In support of these results, the 5′ RACE analysis using the total RNA of phytoplasma-infected plants showed that the upstream region of the major TSS of *tengu* and *PAM486* contained typical RpoD_OY_-dependent promoter elements (Fig. 4). Therefore, RpoD_OY_ seems to mediate transcription of not only several housekeeping genes as the principal sigma factor, which is in agreement with classical theory, but also genes highly expressed in either plant or insect hosts.

How RpoD_OY_-dependent genes such as *tengu* and *PAM486* are highly expressed in plants is still uncertain, despite the fact that *rpoD*_*OY*_ is highly expressed in insects. We propose two hypotheses to explain these observations. First, other phytoplasma transcriptional regulators may positively or negatively affect the gene expression levels. For example, histone-like protein (HimA) conserved in phytoplasmas is one of the candidate transcription regulators. In some bacteria, histone-like proteins act as a transcriptional repressor by binding to DNA^44^,^45^. When phytoplasmas infect insect hosts, HimA may repress the expression of some of the RpoD_OY_-dependent genes, which leads to their specific expression in plant hosts. Second, non-coding RNAs may play roles as gene regulatory factors. Recent studies have revealed that non-coding RNAs such as riboswitches and small RNAs regulate their gene expression in many bacteria^46^,^47^. In a previous study, we used 5′ RACE analysis to show frequent transcription initiation within the coding regions of genes^48^. We found in this study, using *in silico* promoter analysis, many promoter elements that positioned intergenic regions far from the ATG initiation codon or within the coding regions of genes, implying the existence of many non-coding RNAs in phytoplasmas.

In class *Mollicutes*, mycoplasmas and spiroplasmas possess *rpoD* as a single sigma factor gene in their genomes^49^,^50^. Like phytoplasmas, however, many mycoplasma genes are differentially expressed under various conditions^51^,^52^, and several spiroplasmas multiply in distinct plant and insect hosts^53^. Further studies of these bacteria will provide insight into the gene expression mechanisms of phytoplasmas.

Our *in silico* analysis detected 88 RpoD_OY_-dependent genes in the OY-M genome. Some of these genes, such as *znuA*, rRNA-16S, and tRNA-Glu, were known as the first genes of operons that could be regulated by the principal sigma factor in other bacteria^21^,^22^,^54^,^55^. In phytoplasmas, operon structures initiated from these genes are also conserved. Therefore, given the presence of these and other RpoD_OY_-dependent operons, RpoD_OY_ is likely to regulate more than 88 genes identified by our *in silico* analysis, even though additional studies are needed to determine whether each mRNA of putative RpoD_OY_-dependent genes is monocistronic or polycistronic.

Numerous studies have implied that the principal sigma factor gene is transcribed by the RNAP holoenzyme containing the principal sigma factor itself  56,57,58. However, in *Streptomyces griseus*, the principal sigma factor *hrdB* is not controlled by itself, but by the alternative sigma factor ShbA, which is known as an ECF sigma factor^59^. The ECF-subfamily sigma factors, the activity of which is regulated at the post-transcriptional level, usually activate the transcription of specific genes in response to environmental changes^60^. Although our results suggested a role of RpoD_OY_ as the principal sigma factor, P*rpoD* was not controlled by RNAP_Ec_–RpoD_OY_ (Fig. 3). The possibility exists that *rpoD*_*OY*_ is transcribed as polycistronic mRNAs, but we could not find the upstream genes of *rpoD*_*OY*_ (e.g., the PAM624 and glycyl-tRNA synthetase genes) that can constitute a set of operons containing *rpoD*_*OY*_ from the list of putative RpoD_OY_-dependent genes (see supplementary Table S1 online). These findings imply that *rpoD*_*OY*_ transcription is regulated by a sigma factor other than RpoD_OY_, such as the putative ECF-like sigma factor FliA_OY_, raising the intriguing possibility that the sigma factor that transcribes *rpoD*_*OY*_ may play a key role as an environmental sensor because *rpoD*_*OY*_ expression is altered through host switching. Further studies are necessary to assess the effects of transcription activity of other transcriptional regulators such as FliA_OY_ using *in vitro* transcription assays, which can reveal the detailed infection mechanisms of phytoplasma host switching between plants and insects.

## Materials and Methods

### Preparation of phytoplasma-infected plants and insects

The ‘*Ca*. P. asteris’ OY strain (OY) was isolated in Saga Prefecture, Japan^61^. A derivative line of OY (OY-M) was maintained in garland chrysanthemum (*Chrysanthemum coronarium*) using the leafhopper vector insect *Macrosteles striifrons*^62^. Plants infected with OY-M produce many lateral shoots, but exhibit only mild leaf yellowing and almost no stunting. OY-M-infected host plants exhibiting typical symptoms were maintained at 25 °C in a greenhouse with a 16-h light/8-h dark photoperiod until use for analysis. For total RNA extraction from insects, OY-M-carrying leafhoppers that fed on OY-M-infected plants for 40 days were used.

### 5′ RACE analysis and promoter prediction

To identify the 5′-end of mRNA in OY-M, 5′ RACE analysis was performed using the 5′ RACE System for Rapid Amplification of cDNA Ends (Life Technologies Inc.) according to the manufacturer’s protocol. Polymerase chain reaction (PCR) amplification was accomplished using Taq DNA polymerase (TaKaRa Bio Inc.), a nested gene-specific primer (RACE1 or RACE2) that anneals to a site located within the cDNA molecule (see Supplementary Table S1 online), and an anchor primer (Life Technologies Inc.). The nested PCR products were visualized by agarose gel electrophoresis and cloned using the pCR 2.1 TOPO® TA Cloning® Kit (Life Technologies, Inc.). Nucleotide sequences were determined by the dideoxynucleotide chain-termination method using an ABI Prism® 3130 Genetic Analyzer (Applied Biosystems). We sequenced at least eight independent clones and defined the most common 5′-end as a putative transcriptional start site of the genes. The consensus of the OY-M gene promoters was predicted using BioProspector^63^, available at http://ai.stanford.edu/~xsliu/BioProspector/. The resulting consensus promoter sequence was illustrated based on multiple sequence alignments using the WebLogo tool^64^, available at http://weblogo.berkeley.edu/.

### Plasmid construction and expression of His-RpoD_OY_

To construct pCold_His-RpoD_OY_, the full-length *rpoD* gene of OY-M was PCR-amplified using a primer pair *rpoD*_kpnF (5′-GGG GTA CCA TGG AAT TCG ATA ACA TAA TCA AAA -3′) and *rpoD*_salR (5′-CGA CGT CGA CTT ATT TGT GGT TGT GGT ACA AAC TTT TT-3′). The amplified DNA fragments were digested with *Kpn*I and *Sal*I, followed by cloning into pColdI (TaKaRa Bio Inc.) digested with the same enzymes. The resulting plasmid was subsequently transformed into *E. coli* strain BL21-CodonPlus™ (DE3)-RIL cells (Stratagene). The transformed *E. coli* was precultured at 37 °C in lysogeny broth (LB) medium containing ampicillin (50 μg/ml). The overnight culture (2 ml) was added to 100 ml LB medium containing ampicillin (50 μg/ml). After incubation for 1 h at 37 °C and subsequent incubation for 30 min at 15 °C, protein expression was induced by the addition of 0.1 mM isopropyl-β-D-thiogalactopyranoside (IPTG). Cells were cultured at 15 °C for 24 h following induction.

### *In vitro* transcription assay

N-terminally His-tagged-RpoD_OY_ was purified as previously described^65^ after overexpression in *E. coli* from pCold_His-RpoD_OY_. A DNA-dependent RNA polymerase (RNAP) holoenzyme containing RpoD_OY_ (RNAP_Ec_-RpoD_OY_) was made by adding 20 ng of purified RpoD_OY_ to 1 U of *E. coli* RNAP core enzyme (Epicenter) in buffer [50 mM Tris–HCl, 100 mM KCl, 10 mM MgCl_2_, 1 mM dithiothreitol, 0.1 mM EDTA, 5% (v/v) glycerol, pH 8.0] overnight at 4 °C. The reconstituted complex was verified by NativePAGE™ gel electrophoresis (Life Technologies Inc.; see Supplementary Fig. S4 online). Transcription was initiated by adding 22 μl of NTP mix (500 μM ATP, 500 μM UTP, 500 μM GTP, 40 μM CTP; TaKaRa Bio Inc.), 2 μl of [alpha-32P]CTP (800 Ci/mmol; PerkinElmer), and 800 ng of DNA template to the reconstituted RNAP holoenzyme under the same buffer conditions. DNA fragments used for a template were amplified by PCR using the primer pair (see Supplementary Table S2 online) and purified using an UltraClean® 15 DNA Purification Kit (MO BIO Laboratories). A series of substitution templates were obtained *via* a recombinant PCR method^66^. Reaction mixtures were incubated for 15 min at 37 °C, and reactions were stopped by incubation for 5 min at 80 °C. Samples were treated with DNaseI for 15 min, followed by phenol–chlorophorm extraction and ethanol precipitation, and resuspended in 15 μl loading dye containing 7.5 M urea. Purified transcripts were analyzed using 6% (w/v) polyacrylamide–7 M urea gel electrophoresis and autoradiography. Signal intensities from autoradiographs were determined with the FLA-5000 image reader (GE Healthcare). All experiments were repeated at least twice and consistent results were obtained among replicates.

### *In silico* prediction of RpoD_OY_-dependent genes in phytoplasma genomes

Promoter predictions were made in the genomes of ‘*Ca*. P. asteris’ OY-M, AYWB, ‘*Ca*. P. australianse’, and ‘*Ca*. P. mali’ AT (GenBank Accession Numbers AP006628.2, CP000061, AM422018, and CU469464, respectively) using genome sequence files obtained from the NCBI genome database (http://www.ncbi.nlm.nih.gov/genome/ ). Whole genome sequences were searched for promoter sequence patterns using ‘dna-pattern’ of RSA-tools (http://rsat.ulb.ac.be/index.html). The resulting sequences were matched to each reference genome sequence, and the genes to which the resulting sequences were matched within 500 bp upstream of either ATG or 5´-end of mature rRNA and tRNA are listed in Supplementary Table S3 online.

## Additional Information

**How to cite this article**: Miura, C. *et al.* Functional characterization of the principal sigma factor RpoD of phytoplasmas *via* an *in vitro* transcription assay. *Sci. Rep.* **5**, 11893; doi: 10.1038/srep11893 (2015).

## Supplementary Material

Supplementary Information
