# Supplementary material for: Functional characterization of the principal sigma factor RpoD of phytoplasmas via an in vitro transcription assay
Source: Sci Rep. 2015 Jul 7;5:11893. doi: 10.1038/srep11893 (PMC4493692; doi:10.1038/srep11893)
Supplement: Supplementary Information [file srep11893-s1.pdf]

Supplementary information

Functional characterization of the principal sigma factor RpoD of phytoplasmas *via* an *in vitro* transcription assay.

Chihiro Miura[1], Ken Komatsu[2], Kensaku Maejima[1], Takamichi Nijo[1], Yugo Kitazawa[1], Tatsuya Tomomitsu[1], Akira Yusa[1], Misako Himeno[1], Kenro Oshima[1], & Shigetou Namba[1]*

[1]Graduate School of Agricultural and Life Sciences, The University of Tokyo, 1-1-1 Yayoi, Bunkyo-ku, Tokyo, 113-8657, Japan.

[2]Graduate School of Agriculture, Tokyo University of Agriculture and Technology, 3-5-8 Saiwaicho, Fuchu, Tokyo 183-8509, Japan.

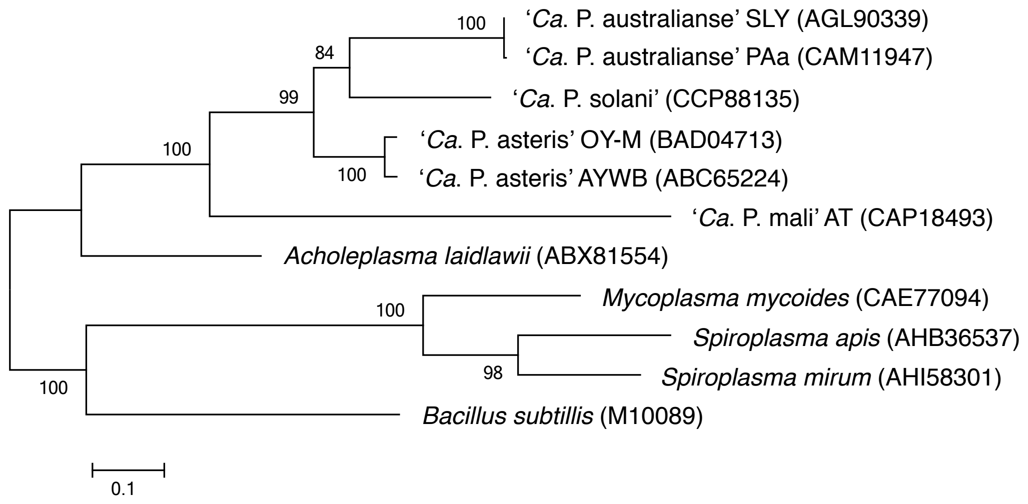

Supplementary Figure S1. Phylogenetic relationships of RpoD

The phylogenetic tree constructed by using neighbor-joining method shows the relationship of six phytoplasma RpoDs ['*Candidatus*. Phytoplasma. australianse' strains SLY and PAa, '*Ca*. P. solani', '*Ca*. P. asteris' strains OY-M and AYWB, and '*Ca*. P. mali' strain AT], five other mollicutes species, and *Bacillus subtilis* as an outgroup. Numbers in parentheses indicate accession numbers.

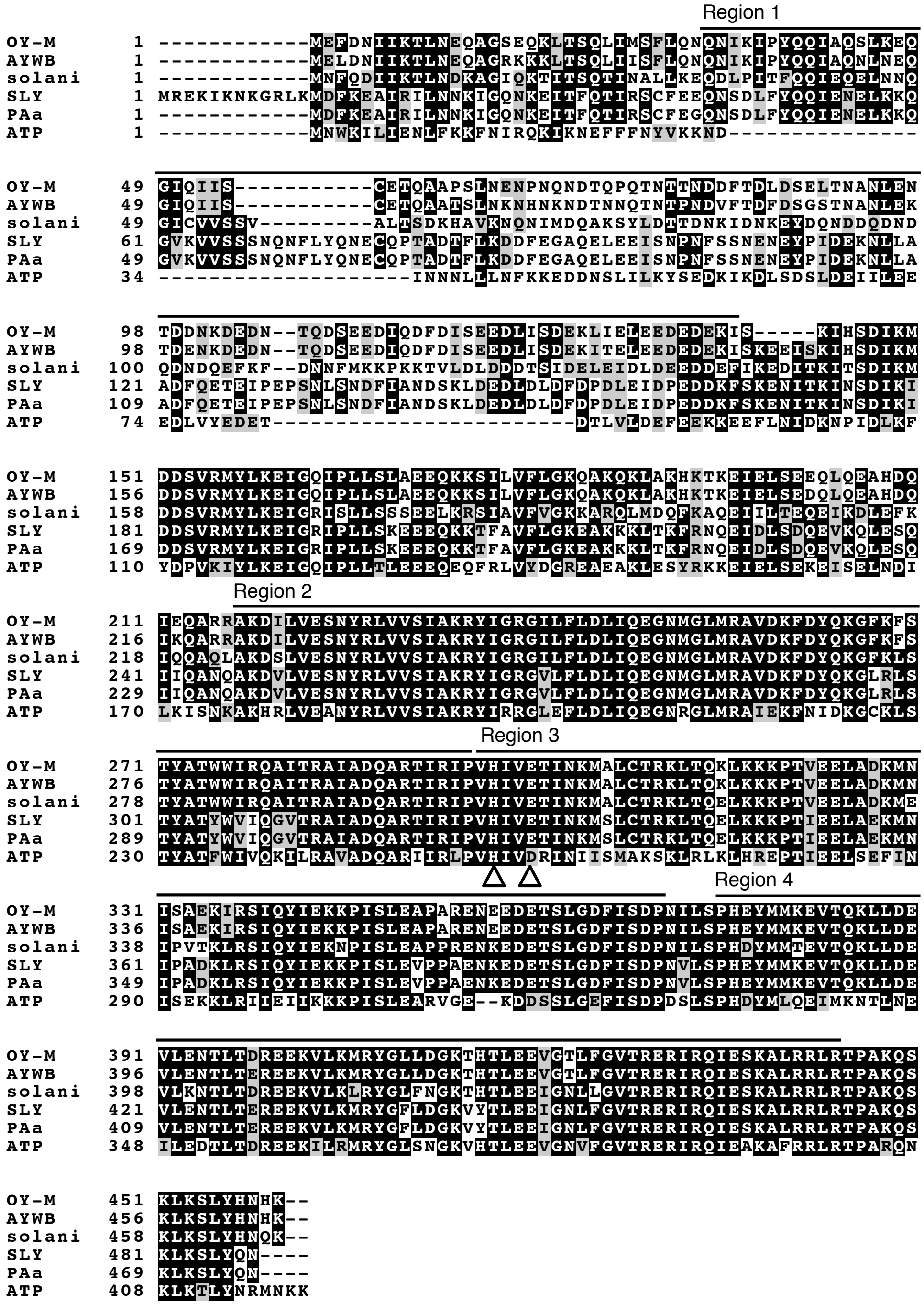

Supplementary Figure S2. Amino acid alignment of RpoD

The amino acid sequences of RpoDs of six phytoplasmas (OY-M, '*Ca*. P. asteris' strain OY-M; AYWB, '*Ca*. P. asteris' strain AYWB; solani, '*Ca*. P. solani'; SLY, '*Ca*. P. australianse' strain SLY; PAa, '*Ca*. P. australianse' strain PAa; and ATP, '*Ca*. P. mali' strain AT) were aligned using the CLUSTAL W program (http://www.genome.jp/tools/clustalw/), and edited with BoxShade (http://www.ch.embnet.org/software/BOX_form.html). Black and gray boxes indicate ≥ 50% identical and similar amino acid residues, respectively. Arrowheads indicate the positions of histidine and glutamic acid residues, which are conserved among bacterial sigma factors.

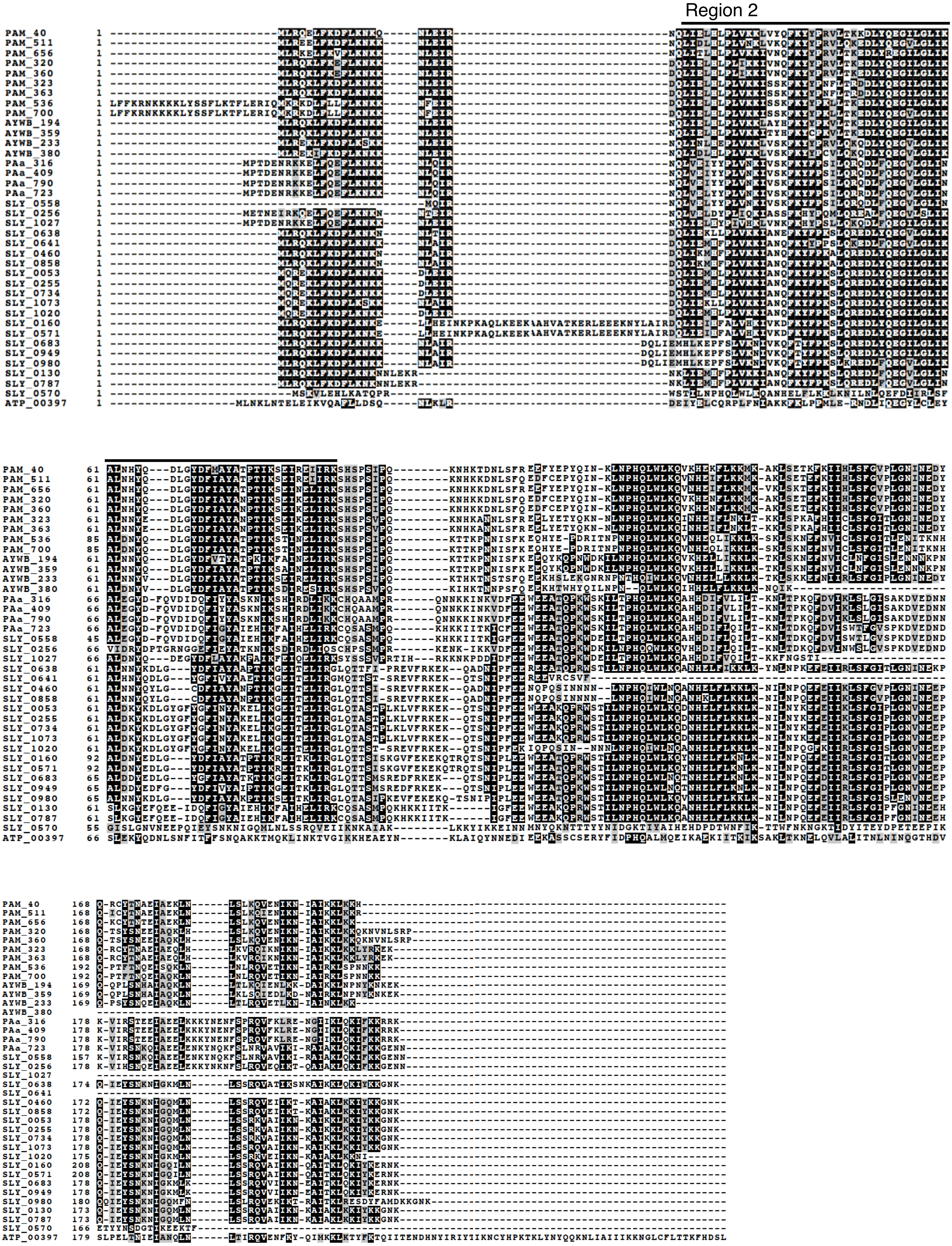
Region 2

Supplementary Figure S3. Amino acid alignment of FliA

The amino acid sequences of 38 FliAs of five phytoplasmas (PAM, '*Ca*. P. asteris' strain OY-M; AYWB, '*Ca*. P. asteris' strain AYWB; PAa, '*Ca*. P. australianse' strain PAa; SLY, '*Ca*. P. australianse' strain SLY; and ATP, '*Ca*. P. mali' strain AT) were aligned and edited as described above. Black and gray boxes indicate ≥ 50% identical and similar amino acid residues, respectively.

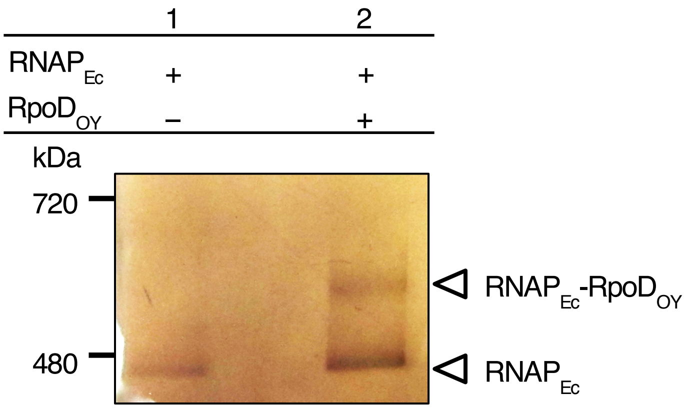

Supplementary Figure S4. Reconstituted RNAP holoenzyme

RNAP$_{Ec}$ was incubated without (lane1) or with (lane2) RpoD$_{OY}$ in reconstitution buffer. Arrowheads indicate the positions of RNAP$_{EC}$ (378 kDa) and RNAP$_{EC}$–RpoD$_{OY}$ complex (about 550 kDa), respectively.

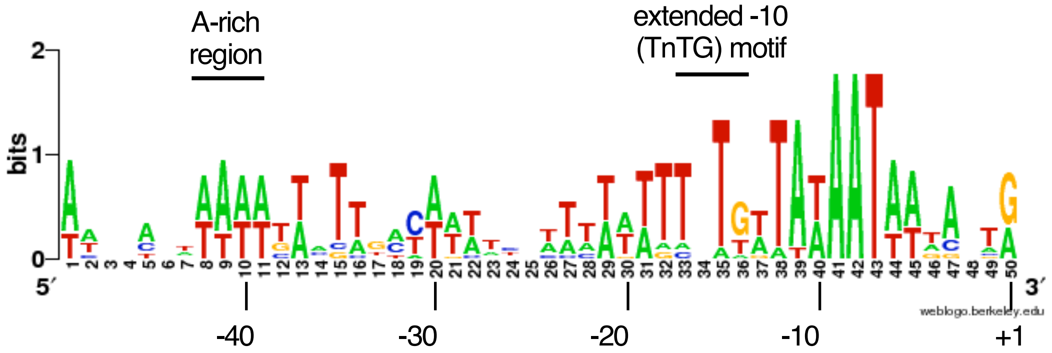

Supplementary Figure S5. Sequence conservation in the RpoD$_{OY}$-dependent promoter region

Sequence logo derived from the alignment of the 11 defined promoter regions shows a semi-conserved extended –10 motif (5'-TnTG-3') positioned around –17 to –14 and A-rich region positioned around –42 to –39.

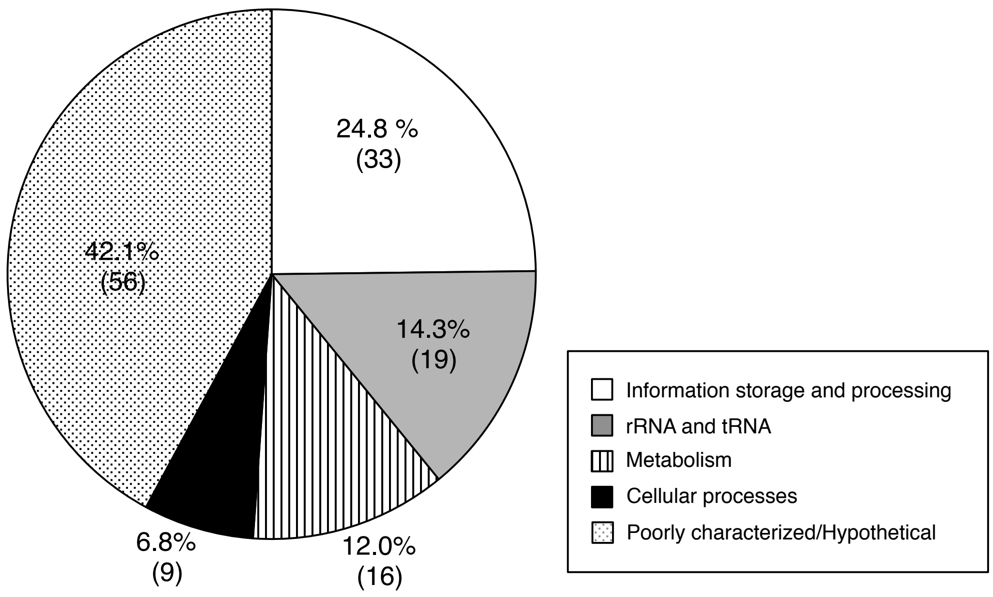

Supplementary Figure S6. COG distribution of putative $RpoD_{OY}$-dependent genes

Putative $RpoD_{OY}$ dependent genes were categorized into Clusters of Orthologous Groups (COGs), and presented as pesentages. The number inside the brackets represents the number of genes in each category.

Supplementary Table S1. Primers used in 5' RACE analysis

primer	5'→3'
*rrnB*_RACE1	TCATAGTCTTGGTAGGCC
*rrnB*_RACE2	CGTTACTCACCCGTTCGCC
*ibpA*_RACE1	CAAGATAATCAATACTTTATTACTATTGAATTACCAG
*ibpA*_RACE2	CTGGTAATTCAATAGTAATAAAGTATTGATTATCTTG
*infC*_RACE1	CTTTTAAAACAATAATTTGGGTTTTCTTTTTGGC
*infC*_RACE2	CTAATGAATTATCAAAAACATCGTTATAACCAA
*rplM*_RACE1	ACAACAATAACATAATCGCCATTGTCTACG
*rplM*_RACE2	GTGTTTTCCTTTTAAAATGGAAGCCACTTTGG
*rpsD*_RACE1	CCGTGATTAGCTAATTGTCTTGCTTGAGCTC
*rpsD*_RACE2	CCTAAACGATAAACTACATTATCAAGACGAGATTC
*PAM157*_RACE1	AAATATTTGTTGCTGTTTGTTGCC
*PAM157*_RACE2	TTTGGGTTTGGTGTGATTTC
*amp*_RACE1	TGAACTTTCCATTGTTTAACAACTTTTTCTGC
*amp*_RACE2	AGCAGCAGTAAGTTCAAGAGCGTC
*PAM289*_RACE1	AAATCGTCTCCAATAGAAAGACC
*PAM289*_RACE2	AAGTAATAATGCACTTAAACAATAAATAAACC
*PAM486*_RACE1	CCCAATAATCAACTCCTAGAACGCT
*PAM486*_RACE2	ATTTTGTAAGCCACGCAAGAACATG
*tengu*_RACE1	AAATTAGGCATCTTTCTCGCC
*tengu*_RACE2	TTTAGTTTCAATTAGAGTTATCACG

Supplementary Table S2. Primers used in the *in vitro* transcription assay

primer	5’→3’
P*rrnB*_F	TTGCCGGAATTCAATCTTAAAAC
P*rrnB*_R	TTACAAAATTGATGCATCAAAAATTGC
P*rrnB*_P2_F	TTAACAAAAGTATTTTTTTACTTGCCAAATCCT
P*rrnB*_P2_R	same as *rrnB*_RACE_1
P*ibpA*_F	GTTTGGTTATTTTCATGTAATTTATCAAAATATAAGG
P*ibpA*_R	CTGGTAATTCAATAGTAATAAAGTATTGATTATCTTG
P*infC*_F	AGTTTTGCAAAAAAAATAACCTCTTAATTGCC
P*infC*_R	CTTTTAAAACAATAATTTGGGTTTTCTTTTTGGC
P*rplM*_F	TATTTGCCAGACCAAAAAGCTGTTATTAGTG
P*rplM*_R	GTGTTTTCCTTTTAAAATGGAAGCCACTTTGG
P*rpsD*_F	AATTACAACCTCAAACTATTTACTTAGTTTGGGG
P*rpsD*_R	CCGTGATTAGCTAATTGTCTTGCTTGAGCTC
P*rpoD*_F	AGTTGTTCTCCTAAGACTTCACTTC
P*rpoD*_R	CCTTTCTAAAAATATTTTTAGAAAAGATAGGAA
P*157*_F	TGCTTGGAATATGTAGCAATTACTGAGATTATAAAAAG
P*157*_R	AAATAATGTTCAATAATGTTTGTCTTGGTATCGGG
P*289*_F	CACAAACACAAAATAAAATGTAAGCGTAGA
P*289*_R	AAGTAATAATGCACTTAAACAATAAATAAACC
P*486*_F	TAGAAAATAAATAAATTTTAAAAAT
P*486*_R	ATTTTGTAAGCCACGCAAGAACATG
P*tengu*_F	AAACCGTTTTAAAATGACCTGAATT
P*tengu*_R	TTTAGTTTCAATTAGAGTTATCACG
P*amp*_F	AGAAAATAAAGATAATAACTTTGATTTAGGAACACAAG
P*amp*_R	same as *amp*_RACE2

Supplementary Table S3. List of predicted $RpoD_{OY}$-dependent genes

No.	PAM_No.	Gene	-35	-10	Homologous genes of other phytoplasmas
1	*PAM008*	*rfaG*	TTGCTT	TATAAA	ATP[†]_00083
2	*PAM011*	*rpsR*	CTGATA	TAAAAT	
3	*PAM022*	*dppB*	CAGATT	TAAAAT	
4	*PAM023*	*hyporhetical*	CTGATA	TAAAAT	
5	*PAM028*	*ibpA*	TTGACA	TAAAAT	PAa[††]_0717, AYWB[†††]_017
6	*PAM033*	*hyporhetical*	TTGCTA	TATAAA	
7	*PAM046*	*hyporhetical*	CTCATT	TAAAAA	
8	*PAM059*	*mdlB*	TTCCTT	TAAAAT	
9	*PAM060*	*hyporhetical*	TTCATA<br>TACCCT	TATAAT<br>TATAAT	
10	*PAM063*	*himA*	TAGATT	TATAAT	
11	*PAM070*	*hyporhetical*	TAGATA	TATAAT	
12	*PAM078*	*artM*	TAGATT	TAAAAT	
13	*PAM081*	*rpsT*	TTGCTT	TATAAT	AYWB_633
14	*PAM083*	*serS*	TACATT	TAAAAT	
15	*PAM085*	*hyporhetical*	TTCCTA	TAAAAT	AYWB_630
16	*PAM092*	*znuA*	TTCATA	TATAAT	ATP_00492, PAa_0481, AYWB_624
17	*PAM106*	*rpmB*	CACCCA	TATAAT	ATP_00278
18	*PAM109*	*hyporhetical*	TTGACA<br>TAGCTA<br>TTGCTT	TAAAAT<br>TATAAA<br>TATAAA	
19	*PAM120*	*groES*	TTGCCA	TATAAT	ATP_00184, PAa_0760
20	*PAM122*	*amp*	CTCCCA	TAAAAT	
21	*PAM128*	*aspS*	CTCATA	TATAAT	
22	*PAM130*	*hflB*	CTCCTT	TATAAT	ATP_00146, ATP_00460, AYWB_187
23	*PAM138*	*rplM*	TTGACT<br>TACACA	TAAAAT<br>TAAAAA	ATP_00332, PAa_0496, AYWB_582
24	*PAM152*	*pth*	TAGCTA	TAAAAT	PAa_0610
25	*PAM153*	*hyporhetical*	TTGACA<br>TTGCTA<br>TTGCTA	TAAAAT<br>TATAAA<br>TAAAAT	AYWB_566

Supplementary Table S3. ***continued***

No.	PAM_No.	Gene	-35	-10	Homologous genes of other phytoplasmas
26	*PAM164*	*dut*	TTGATT	TATAAT	AYWB_555
27	*PAM198*	*rpmF*	TTGACA	TAAAAT	PAa_0589, AYWB_525
28	*PAM199*	*rpsJ*	TTCATT	TATAAA	
29	*PAM247*	*rpmH*	TTGACA	TATAAT	ATP_00378, AYWB_474
30	*PAM271*	*hyporhetical*	TACCTA	TAAAAT	
31	*PAM274*	*hyporhetical*	CACCCA<br>CTGATA	TAAAAT<br>TAAAAT	
32	*PAM284*	*eno*	TACATT	TATAAT	AYWB_437
33	*PAM288*	*hyporhetical protein*	TAGCCT	TATAAA	ATP_00462, AYWB_433
34	*PAM305*	*alaS*	TTCACT	TATAAT	ATP_00248
35	*PAM309*	*lplA*	TTGATT	TATAAA	
36	*PAM312*	*hyporhetical*	CTGCTT	TATAAT	
37	*PAM326*	*hflB*	TTCCTA	TAAAAT	ATP_00146, ATP_00460, AYWB_187
38	*PAM331*	*hyporhetical*	CAGATT	TAAAAT	
39	*PAM338*	*hyporhetical*	TTCCTA	TAAAAT	
40	*PAM355*	*uvrD*	TTCATT	TATAAA	PAa_0693, AYWB_085
41	*PAM366*	*hflB*	TTCCTA	TAAAAT	ATP_00146, ATP_00460, AYWB_187
42	*PAM371*	*hyporhetical*	CAGATT	TAAAAT	
43	*PAM382*	*hyporhetical*	TTGATA	TAAAAT	
44	*PAM398*	*hyporhetical*	TTCCCT	TAAAAT	
45	*PAM404*	*hyporhetical*	TTGATA	TAAAAT	
46	*PAM406*	*tra5*	TTGATA<br>TTGCCT	TATAAT<br>TATAAA	
47	*PAM413*	*uvrD*	TTGCTT<br>CTGATT	TATAAT<br>TAAAAT	PAa_0693, AYWB_085
48	*PAM433*	*uvrC*	TTGATT	TATAAT	
49	*PAM446*	*hyporhetical*	TTGATA	TAAAAT	
50	*PAM447*	*tldD*	TTGCCT	TATAAT	PAa_0703, AYWB_318
51	*PAM453*	*srmB*	TTCCTT<br>TTCCCA	TATAAT<br>TATAAA	AYWB_318

Supplementary Table S3. *continued*

No.	PAM_No.	Gene	-35	-10	Homologous genes of other phytoplasmas
52	*PAM476*	*mutT*	TAGCCA	TATAAT	
53	*PAM479*	*hyporhetical*	TTGCTT	TATAAT	
54	*PAM486*	*hyporhetical*	TAGATA	TATAAT	
55	*PAM492*	*hyporhetical*	TACCTA	TAAAAT	
56	*PAM509*	*hyporhetical*	CTGCTTG	TATAAT	
57	*PAM526*	*hyporhetical*	TAGACT	TATAAT	
58	*PAM542*	*hyporhetical*	TAGATA	TATAAT	
59	*PAM556*	*hyporhetical*	CTGCTT	TATAAT	
60	*PAM577*	*hyporhetical*	CAGATT	TAAAAT	
61	*PAM586*	*rpsD*	TTGACT	TAAAAT	PAa_0088, AYWB_241
62	*PAM599*	*phnL*	TTGCCT<br>TTGCTT	TATAAA<br>TATAAT	ATP_00101, PAa_0530
63	*PAM600*	*acoA*	TACATT	TATAAA	ATP_00156, AYWB_136
64	*PAM607*	*plsX*	TTCCTT<br>TTCATT	TAAAAT<br>TATAAA	AYWB_142
65	*PAM612*	*psd*	CAGCCT	TATAAT	
66	*PAM618*	*hsdR*	TTGCTA	TATAAA	
67	*PAM632*	*hyporhetical*	TACATT<br>TTGCCT	TATAAT<br>TATAAA	
68	*PAM634*	*hyporhetical*	TTGATA	TATAAA	AYWB_100
69	*PAM635*	*hyporhetical*	TTGACT	TATAAT	
70	*PAM653*	*hyporhetical*	TTCCTA	TAAAAT	
71	*PAM663*	*hyporhetical*	CACCCA	TAAAAT	AYWB_091
72	*PAM664*	*rpt1*	TTCATT	TATAAA	
73	*PAM670*	*hyporhetical*	TTGATA	TAAAAT	
74	*PAM676*	*exo*	TTCATT	TAAAAT	
75	*PAM682*	*hyporhetical*	TACCTA	TAAAAT	
76	*PAM690*	*hyporhetical*	TACACA	TAAAAT	
77	*PAM712*	*hyporhetical*	TAGACA	TATAAA	
78	*PAM719*	*hyporhetical*	TAGCTA	TATAAA	
79	*PAM727*	*hflB*	TAGATT	TATAAT	ATP_00146, ATP_00460, AYWB_187
80	*PAM728*	*hyporhetical*	CAGCTT<br>CAGCTT	TATAAA<br>TAAAAT	AYWB_085

Supplementary Table S3. *continued*

No.	PAM_No.	Gene	-35	-10	Homologous genes of other phytoplasmas
81	*PAM734*	*norM*	CTCCCA	TAAAAT	AYWB_651
82	*PAM741*	*hyporhetical*	TTCATT	TAAAAT	
83	*PAM743*	*rpmI*	CACCCA	TAAAAT	
84	*PAM744*	*infC*	TTGACA	TATAAT	
85	*PAM763*	*uvrB*	TAGATT	TATAAA	
86	*PAM765*	*tengu*	TACATT	TATAAT	
87	*PAMr006*	*rRNA-16S* *ribosomal RNA*	TTGCTA TTGCCA	TATAAT TATAAT	ATP_r0001, PAa_r02, AYWB_r01, AYWB_r04
88	*PAMt029*	*tRNA-Glu*	TTGACT	TATAAT	PAa_t17, AYWB_t04

†, '*Ca*. P. mali'; ††, '*Ca*. P. australianse'; †††, '*Ca*. P. asteris' AYWB
